# Remarks on National Education

**Published:** 1847-01

**Authors:** 


ART. XXI.—*Remarks on National Education.* By GEORGE COMBE.—*Edinburgh,* 1847. 8vo, pp. 33.

ALTHOUGH the question to which these Remarks relate is one which it would be foreign to our purpose to discuss, yet we cannot pass Mr. Combe's pamphlet without notice; since it presents within a narrow compass a large mass of well-directed argument in favour of the position that the State ought not only to provide for, but to compel, the education of the people. He combats with great earnestness the prevalent idea that religion can only be taught dogmatically, and that an education into which it is not formally introduced is thereby "godless;" and points out very clearly that any instruction which tends to enforce those laws on which the Creator has framed our physical and moral constitution, is essentially and directly religious. "The moral and religious sentiments," he truly says, "are the grand levers of civilized society. He who commands them is irresistible; and until science shall discover her own character and vocation, that she is the messenger of God, speaking directly to these sentiments in strains calculated to thrill and rouse them to the most energetic action, she will never wield her proper influence over society for the promotion of their moral, religious, and physical welfare. Never, until she does so, will she take that place in social esteem and veneration, which, as the fountain of divine wisdom, she is entitled to possess."

